# An Unusual Clinical Presentation of New-Onset Herpetic Necrotizing Sclerokeratitis After Implantable Collamer Lens Surgery: A Case Report

**DOI:** 10.7759/cureus.86985

**Published:** 2025-06-29

**Authors:** Heba M Alsharif, Rahaf M Alruwaili, Ahmed Alsaleh

**Affiliations:** 1 Ophthalmology, King Khaled Eye Specialist Hospital, Riyadh, SAU

**Keywords:** corneal melt, herpetic, keratitis, necrotizing scleritis, scleritis

## Abstract

We report a case of atypical new-onset herpetic necrotizing sclerokeratitis after implantable collamer lens surgery. A 37-year-old healthy Saudi female, known to have keratoconus underwent keratoplasty 14 years ago, followed by implantable collamer lens surgery (ICL) for visual rehabilitation nine months ago in the left eye. She presented to our institute with a history of subacute painful ocular pain and redness in her left eye over seven months. Upon assessment, the left eye's visual acuity was 20/200. Examination of the anterior segment showed injected conjunctiva, episcleral and scleral blood vessels, scleral thinning associated with peripheral keratitis, keratic precipitates, and decreased corneal sensation. Diagnostic ultrasonography (B-scan) revealed mild vitreous opacities with a normal optic nerve head and no retinal choroidal thickening. The results of the right eye examination were unremarkable. The patient was found to have atypical herpetic necrotizing sclerokeratitis and was treated with topical and oral antivirals for three months, with significant improvement. Thus, timely detection and treatment were crucial for minimizing vision-threatening complications.

## Introduction

Herpes virus-induced ocular infection is a major cause of ocular morbidity [1]. Herpes simplex virus (HSV) can produce a wide range of ocular presentations, ranging from milder conditions as epithelial keratitis to more severe forms like keratouveitis and necrotizing scleritis. Additionally, while herpetic eye disease is mostly associated with primary infection or reactivation of the virus, it can also occur as a first presentation after intraocular surgeries [2-6]. The reason for this is that surgical procedures may breach corneal integrity or other ocular tissues, which can promote the reactivation of an HSV infection. In the literature, several publications reported the new onset of herpetic keratitis following cataract extraction, lamellar and penetrating corneal transplantation surgeries [2-4,7-10]. While implantable collamer lens (ICL) surgery is generally considered safe and effective for the correction of refractive errors, it has been associated with a range of postoperative complications, including anterior uveitis, cystoid macular edema, pigment dispersion, and, less commonly, Urrets-Zavalia syndrome. However, to our knowledge, the occurrence of herpetic necrotizing sclerokeratitis following ICL implantation appears to be rarely reported. Although herpetic reactivation is a recognized complication following intraocular procedures, the development of sclerokeratitis due to herpes simplex virus in the context of phakic intraocular lens implantation remains undocumented in the literature. Accordingly, this case underscores the importance of maintaining clinical vigilance for atypical HSV presentations and initiating prompt antiviral therapy to mitigate complications and optimize patient outcomes.

## Case presentation

**Figure 1:**
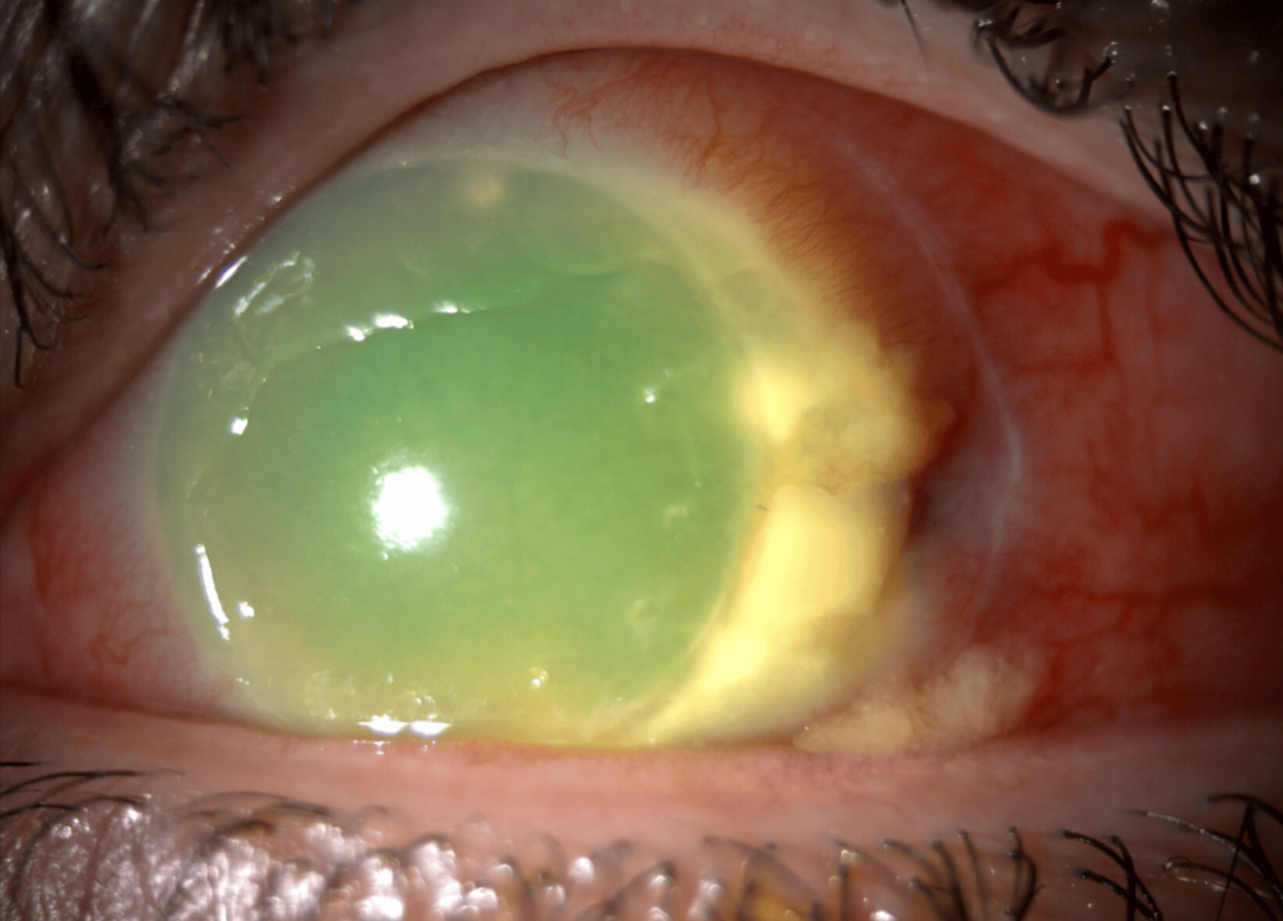
Slit-lamp photo of the left eye showing corneal edema, a subtotal corneal epithelial defect with scleral thinning and peripheral stromal infiltrate temporally.

**Figure 2:**
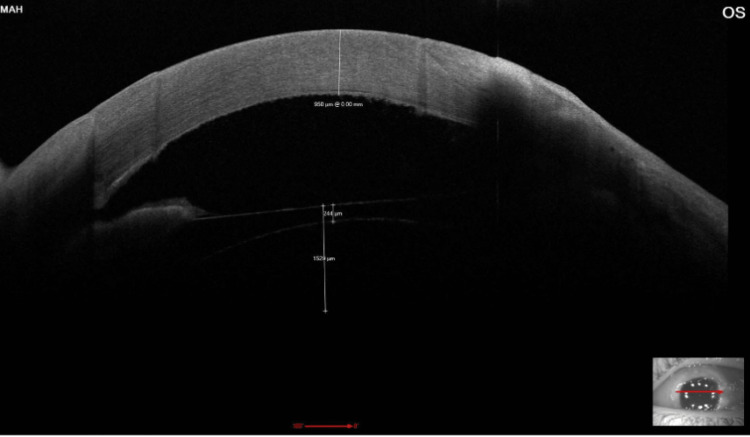
High resolution AS-OCT of the left eye showing corneal thickening measuring 950 microns with mild scleral thinning over the temporal side. AS-OCT: anterior segment ocular coherence tomography

A 37-year-old healthy Saudi female, status post implantable collamer lens (ICL) implantation in the left eye nine months prior to presentation, presented to the emergency department with a history of gradual-onset ocular pain and redness that began seven months ago and increased in intensity over the past two weeks. She has a history of keratoconus and underwent bilateral deep anterior lamellar keratoplasty (DALK) 14 years ago. Her medical and drug history were unremarkable. The family history was not relevant. The patient demonstrated normal intraocular pressure and full extraocular range of motion in both eyes. Visual acuity was preserved in the right eye but markedly reduced in the left (20/200). No proptosis was apparent, though palpation of the globe induced pain in the left eye. The reaction of the pupil showed no afferent pupillary defect in both eyes. Anterior segment evaluation of the left eye using slit-lamp biomicroscopy showed diffusely injected conjunctiva, engorged episcleral and scleral blood vessels, and scleral thinning more pronounced temporally. Moreover, left eye corneal examination showed a subtotal corneal epithelial defect associated with diffuse corneal edema and Descemet’s membrane fold with peripheral whitish infiltrate at the deep layers of cornea extending four clock hours temporally at the host side. Also, it was noted to have diffuse pigmented keratic precipitates (Figures 1, 2). Corneal sensation was diminished in the left cornea in comparison to the right eye. There was no hypopyon in the anterior chamber and a hazy view to assess cell reaction. The iris in the left eye was normal. The ICL was in place with a normal vault without malposition, while the right eye exhibited no abnormalities in the anterior segment, and fundus examination was normal, whereas the left fundus could not be visualized.

The initial ocular workup of the left eye included corneal scraping for stains, cultures, and sensitivity, which was performed twice and yielded negative results with no growth of any organisms. In addition, a corneal swab for herpes simplex polymerase chain reaction (PCR) was taken and yielded negative results. Diagnostic ultrasonography (B-scan) revealed mild vitreous opacities with normal optic nerve head and no retinal choroidal thickening. Ultrasound biomicroscopy showed a normally positioned ICL with thickened anterior sclera mainly temporally. The right eye ocular workup yielded unremarkable results. An extensive uveitis evaluation was carried out, returning negative findings for the tuberculin skin test (purified protein derivative {PPD}), autoimmune markers, including rheumatoid factor, antinuclear antibodies, and antineutrophil cytoplasmic antibodies, as well as viral and infectious screenings for hepatitis, HIV, and syphilis. Imaging studies, comprising a chest X-ray and CT scan, revealed no significant abnormalities. The possibility of ocular sarcoidosis was ruled out by the absence of hilar lymphadenopathy and parenchymal lung lesions on chest CT, in conjunction with a normal serum ACE level.

Initially, the patient was started on broad-spectrum topical fortified antibiotic drops - vancomycin (50 mg/mL) and ceftazidime (25 mg/mL) - administered alternately around the clock to the left eye. In addition to cyclopentolate drops 1% three times daily. During treatment, the patient was not improving symptomatically and clinically, with mild worsening. Thus, based on the collective clinical and investigative findings, a diagnosis of atypical herpetic anterior necrotizing sclerokeratitis was considered, and the patient was subsequently started on topical ganciclovir gel 1.5 mg/g five times daily and oral valacyclovir 1 g three times daily and significant improvement was reported after three days from initiating antiviral regimen.

**Figure 3:**
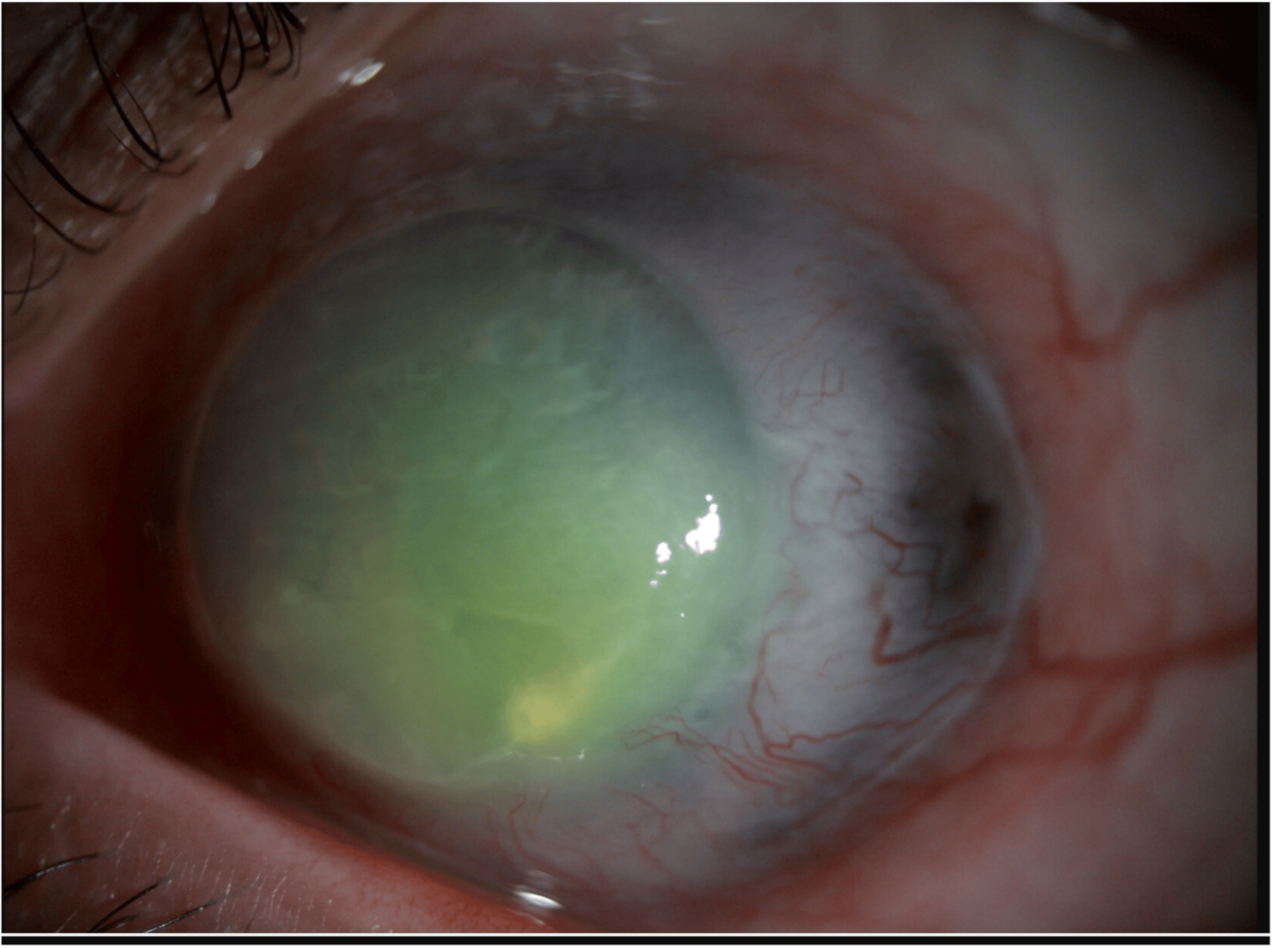
Slit-lamp photo of the left eye four months after treatment, showing reduced conjunctival injection, healed corneal epithelium with mild irregularity, and a temporal thinned staphylomatous area.

One month after receiving the antiviral therapy, the patient showed significant improvement symptomatically and clinically. However, as part of the disease process and given the potential toxic effects of topical antiviral therapy, the patient developed a persistent epithelial defect in the left eye, which was subsequently managed with amniotic membrane transplantation. The patient exhibited complete resolution of the acute attack after four months, but as a sequelae of the inflammatory attack, the patient developed decompensated graft characterized by corneal edema, extensive neovascularization, which yielded to hand motion vision. Nevertheless, the affected scleral thinning area became slightly staphylomatous temporally with no uveal show (Figure 3). She was kept on a prophylactic dose of systemic antiviral therapy.

## Discussion

Necrotizing scleritis is the most destructive and vision-threatening form of scleritis. It is an uncommon, painful ocular condition that causes tissue damage and excruciating ocular pain. Despite the fact that the majority of scleritis cases are caused by immune-mediated diseases, a smaller proportion are caused by infections [5]. Although uncommon, an infectious origin should be taken into account in any patient with scleritis, especially those who have postoperative necrotizing scleritis or persistent, long-lasting inflammation [6]. The commonly reported infectious causes are bacterial, such as Pseudomonas species, tuberculosis, syphilis, and viral, such as herpes zoster and herpes simplex virus [5]. It has been estimated that 149 people per 100,000 have ocular HSV prevalence [8]. Recent molecular biology studies of human ganglia revealed evidence of HSV infection in up to 94% of specimens from persons over 60 years of age [11]. Moreover, due to the high innervation density of the corneal epithelium, disruption of the corneal nerves may function as a potent trigger for HSV-1 reactivation [12]. Thus, a patient can be at risk for reactivation of HSV following ocular surgeries even in the absence of previous evident infection. Many reports of the new onset of herpetic infection in individuals without prior history of herpetic eye disease have been documented [2-4,7-10]. A study by Borderie et al. presented three patients who underwent penetrating keratoplasty, where two of them experienced keratouveitis and primary graft failure, and the third one experienced dendritic keratitis; all of them had no known history of HSV infection [4]. Moreover, another case series by Rezende et al., Mannis et al., and Remeijer et al. reported the new incidence of epithelial herpetic keratitis ranging from dendritic to geographic ulcers in the graft of individuals who underwent penetrating keratoplasty for reasons other than herpetic infection, where in most of cases, the infection developed within the first two years following the transplant [8-10]. Additionally, other publications by Hu et al., Jhanji et al., and Barequet and Wasserzug presented several cases of epithelial keratitis that developed following cataract and lamellar keratoplasty surgeries in patients with no prior history of ocular viral disease. All cases responded well to antiherpetic medications [2,3,7]. This is a case of herpetic necrotizing sclerokeratitis occurring in an otherwise healthy, immunocompetent patient following ICL implantation for visual rehabilitation after keratoplasty, in the absence of any prior history of ocular viral infection. Although in the present case the diagnosis of herpetic sclerokeratitis was not confirmed by PCR testing of a corneal swab, the diagnosis was made based on the clinical picture and the dramatic response to oral and topical antiviral therapy, in addition to the evident finding of corneal hypoesthesia in the left eye. Therefore, we concluded that viral activation or reactivation should always be considered as a potential cause of atypical postoperative inflammation, especially in refractory cases unresponsive to the typical management.

## Conclusions

This case highlights the need to consider herpetic necrotizing sclerokeratitis as a potential diagnosis in patients presenting with atypical scleritis after implantable collamer lens (ICL) surgery, even in healthy individuals with no prior history of herpetic eye disease. The absence of classic signs can make diagnosis challenging, and delayed recognition may lead to serious, potentially irreversible vision loss. This case serves as a reminder that herpetic disease can occur unexpectedly, and that clinical vigilance is key. Prompt diagnosis and early initiation of antiviral therapy can make a critical difference in outcomes, helping to preserve vision and prevent long-term complications. For both clinicians and patients, this reinforces the importance of careful follow-up and a broad differential when managing unusual postoperative inflammation.

## References

[1] Kaufman HE (2002). Can we prevent recurrences of herpes infections without antiviral drugs? The Weisenfeld Lecture. Invest Ophthalmol Vis Sci.

[2] Hu F, Guan W, Zhang Y, Peng X (2022). Herpetic uveitis caused by herpes simplex virus after cataract surgery in a patient without prior viral keratitis or uveitis: a case report. BMC Ophthalmol.

[3] Jhanji V, Ferdinands M, Sheorey H, Sharma N, Jardine D, Vajpayee RB (2012). Unusual clinical presentations of new-onset herpetic eye disease after ocular surgery. Acta Ophthalmol.

[4] Borderie VM, Méritet JF, Chaumeil C (2004). Culture-proven herpetic keratitis after penetrating keratoplasty in patients with no previous history of herpes disease. Cornea.

[5] Murthy SI, Sabhapandit S, Balamurugan S, Subramaniam P, Sainz-de-la-Maza M, Agarwal M, Parvesio C (2020). Scleritis: differentiating infectious from non-infectious entities. Indian J Ophthalmol.

[6] Gonzalez-Gonzalez LA, Molina-Prat N, Doctor P, Tauber J, de la Maza MT, Foster CS (2012). Clinical features and presentation of infectious scleritis from herpes viruses: a report of 35 cases. Ophthalmology.

[7] Barequet IS, Wasserzug Y (2007). Herpes simplex keratitis after cataract surgery. Cornea.

[8] Rezende RA, Uchoa UB, Raber IM, Rapuano CJ, Laibson PR, Cohen EJ (2004). New onset of herpes simplex virus epithelial keratitis after penetrating keratoplasty. Am J Ophthalmol.

[9] Mannis MJ, Plotnik RD, Schwab IR, Newton RD (1991). Herpes simplex dendritic keratitis after keratoplasty. Am J Ophthalmol.

[10] Remeijer L, Doornenbal P, Geerards AJ, Rijneveld WA, Beekhuis WH (1997). Newly acquired herpes simplex virus keratitis after penetrating keratoplasty. Ophthalmology.

[11] (1998). The Cornea. Second Edition. The Cornea. 2nd ed. Boston: Butterworth-Heinemann.

[12] Beyer CF, Hill JM, Reidy JJ, Beuerman RW (1990). Corneal nerve disruption reactivates virus in rabbits latently infected with HSV-1. Invest Ophthalmol Vis Sci.

